# Does the COVID-pandemic affect the educational and financial inequality in weekly sport participation in the Netherlands?

**DOI:** 10.1080/21582041.2022.2155867

**Published:** 2022-12-26

**Authors:** Malou Grubben, Remco Hoekman, Gerbert Kraaykamp

**Affiliations:** a Department of Sociology, Radboud University Nijmegen, Nijmegen, The Netherlands; b Mulier Institute, Utrecht, The Netherlands

**Keywords:** Sport participation, COVID, educational inequality, financial inequality

## Abstract

This paper explores the impact of the COVID-pandemic on educational and financial inequality in level of weekly sport participation in the Netherlands. Restrictions due to the COVID-pandemic resulted in several barriers for people to continue sport participation. Lower educated people and individuals with financial problems are expected to have relatively few resources to adapt to the COVID restrictions, and therefore, more likely will decrease their level of weekly sport participation. Using high-quality data from the Dutch Longitudinal Internet Studies for the Social Sciences (LISS) panel, we are able to compare individual sport behaviour before and during the COVID-pandemic. Our findings suggest that the level of weekly sport participation of lower educated people and individuals with financial problems decreased more strongly during the COVID-pandemic. This implies that indeed the COVID-pandemic resulted in increasing educational and financial inequality in sport participation. With these results, our study contributes to a body of knowledge on the broader societal impact of COVID on issues of social exclusion. It may also inform policymakers to critically assess and intensify sport promotion policies directed at vulnerable groups in society.

## 1. Introduction

It is a well-known notion that sports participation is beneficial for the individual and for society (Coalter, 2007; Hoekman et al., 2022). Especially, in the recent COVID-pandemic, these advantages are highlighted and brought to the front. For individuals, sports participation amongst others relates to better physical and mental health and an increased number of social connections (Harvey et al., 2018; Li & Siegrist, 2012; Pandey et al., 2015; Schuch et al., 2016). Moreover, it seems that not being sufficiently physically active is related to a higher risk of serious COVID-outcomes after infection (Sallis et al., 2021). In our study, we focus specifically on individual sport participation, a facet of physical activity. Similar to the effect of physical activity, a recent study found that regular sports participation seems to positively affect COVID-outcomes (Halabchi et al., 2021). Societal benefits of sport participation are also reported on increasing social cohesion (Van der Meulen, 2007), lower healthcare costs (Peters & Van der Tuin, 2017) and even reduced crime rates (Bailey et al., 2013; Peters & Van der Tuin, 2017).

The European Union has a long history of policy plans aiming at more sports participation among all groups in society, starting with the European Sport for All charter in 1976 (Council of Europe, 1976). More recently, the United Nations adapted seventeen Sustainable Development Goals. To reach these goals among others stimulating sport for all is perceived as an important tool (United Nations, 2015). Also in the Netherlands, there is long-standing policy to increase sport participation (e.g. Ministerie van VWS, 2018). This policy is mainly executed by local sports managers, and in more recent years, by neighbourhood sports coaches, who try to stimulate people who do not participate in sports (sufficiently) (Hoekman et al., 2017). Despite all efforts and policy intentions, sports participation is still socially stratified in all member states of the European Union, including the Netherlands; women, disabled people, people with a migration background, the lower educated and those with low incomes are less likely to participate in sport (Demarest et al., 2014; European Commission, 2017; Hoekman et al., 2017). In this study, we focus on educational and financial inequality in sport participation. From studies on past global financial crises, we have learned that vulnerable groups in society are likely most affected by crises, intensifying social inequalities (Collins & Haudenhuyse, 2015; Roberts, 2013). Moreover, at the policy level, budgets for ‘Sport for all’ policies, mainly intended for vulnerable groups in society, have decreased during the previous global financial crisis (King, 2013). Based on these findings, we presume the impact of the global health crisis ‘COVID’ on individuals’ daily lives, including sport, work and financial situation, to be greatest among vulnerable groups.

**Figure 1.**
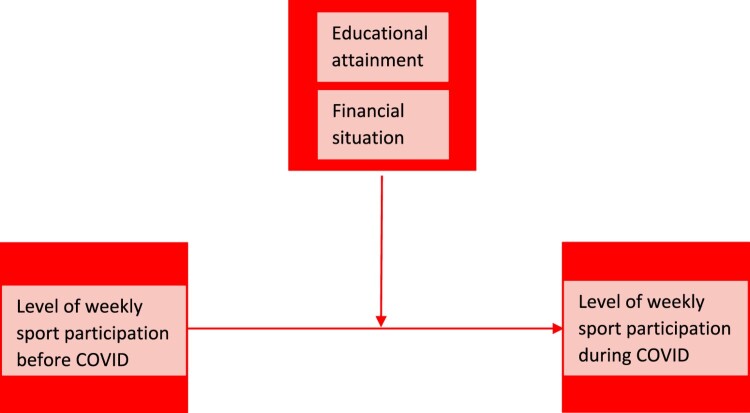
Conceptual model.

Several studies, in the Netherlands and in other European countries, showed that the sports sector was impacted significantly during the global COVID-pandemic. A general reduction in the prevalence of sport participation was observed during the COVID-pandemic (Council of Europe, 2020; Mutz & Gerke, 2021; NOC*NSF, 2020; Tison et al., 2020). From these studies, it largely remained unclear which groups in society are affected most by COVID as most studies reported only national averages for previous years. Without doubt, the COVID-pandemic affected people’s daily life and sports behaviour significantly; due to COVID restrictions, several sports facilities closed down (at all, or in the evenings), and public spaces were less available for sporting (lock down). In general, we expect that especially higher educated and people with limited financial problems would be better equipped to adapt to these COVID-related developments. Contrarily, among vulnerable groups, people have less resources (e.g. knowledge, contacts and money) to adapt to a changing environment, and start, or continue sport participation. Consequently, the current COVID-pandemic may have increased already existing social inequalities in sport participation. This would be in line with prior studies, suggesting that earlier global (financial) crises intensified the social inequality in leisure, including sports (Collins & Haudenhuyse, 2015; Roberts, 2013). This paper addresses this issue and analyses changes in individual weekly^1^ sport participation patterns between 2019 (before the COVID-pandemic) and 2021 (during the COVID-pandemic) in the Netherlands. To do so, we answer the following research question: *To what extent has the COVID-pandemic affected the educational and financial inequality in the level of weekly sport participation in the Netherlands?* A conceptual model visualising our research is presented in Figure 1.

Our study builds on previous research on inequality in sport participation (e.g. Hoekman et al., 2011) and on COVID-related studies on changes in sport participation. Compared to previous studies on inequality in sport participation, we try to make several advancements. First, we investigate social differences in sport participation in the context of a so-called natural experiment: the unexpected evolvement of the COVID-pandemic. This provides us with the opportunity to study how an external shock affects already long-lasting inequalities in society. Second, our study adds to earlier research on the impact of COVID on sport participation. Mutz and Gerke (2021), Bu et al. (2021) and Constandt et al. (2020) conducted their work on the offspring of the COVID-pandemic in 2020, when most countries were in complete lockdown and sports facilities were closed. We utilise data on current sports participation in the spring of 2021 when various sports activities opened up again at least to some extent. This makes the issue of which groups make use of the less restricted sports opportunities even more pressing. Third, our study focusses on intra-personal developments in sport participation. Prior COVID-studies primarily addressed the societal level, providing rather general conclusions on average changes in sports. By looking at changes in sport participation within the individual, we try to gain a better insight into which personal characteristics play a role in dealing with COVID-restrictions. A final strength of our study is that we use rich self-collected data within the representative panel of Longitudinal Internet Studies for the Social Sciences (LISS). We questioned 2.831 respondents aged between 16 and 69 years old between 7 June and 27 July 2021 on their sport participation before and during the COVID-pandemic (March–May 2021). Information is available on an individual’s cultural and economic resources, and various control variables (e.g. gender, age, migration background). We are thus able to compare an individual’s sport participation before and during the COVID-pandemic and analyse the impact of cultural and economic resources on these changes, controlled for other relevant indicators.

## 2. Theoretical background and hypotheses

Several theoretical mechanisms may explain why educational and financial differentiation in sport participation exists. According to Bourdieu, differences in lifestyles, which includes sport participation, between social classes depend on the quantity and type of capital that people possess and on an individual’s class-specific habitus (Bourdieu, 1978, 1984; Kraaykamp, 2002). In this article, we study the association of educational level and financial situation with sport participation levels during COVID pointing to the importance of cultural and economic capital for sports participation. Prior studies have demonstrated that educational level and financial situation do play a prominent role in whether or not to participate in sports (Demarest et al., 2014; European Commission, 2017; Hoekman et al., 2017). In addition to an individual’s sporting habitus, we therefore mainly focus on cultural and economic capital as explanatory factors.

### 2.1. Educational level and sport participation

A relatively low educational degree relates to multiple factors that negatively may affect sports participation. As a starting point, mostly Bourdieu’s notion of cultural capital is used, closely associated with Bourdieu’s concept ‘habitus’. A person’s habitus is generally seen as an internalised system of dispositions that engender all the thoughts, perceptions and actions of an individual (Bourdieu, 1984). Habitus is not solely seen as an individual system as it is developed in interaction with an individual’s social environment. A personal habitus is the product of especially early childhood experiences and socialisation within a person’s social environment (Reay, 2004). According to Bourdieu, an individual’s sporting habitus is class-specific and correlates with differences in sport behaviour (Bourdieu, 1978). Generally, it may be assumed that lower educated people are less socialised with participating in sport as the norm, and therefore, participating in sport is less embedded in their internalised system of dispositions. This may be further explored in several directions.

First of all, lower educated generally are believed to possess less cultural competency and knowledge on healthy behaviour, including the health-promoting effects of participating in sports (André et al., 2018; Rademakers, 2014) . Consequently, lower educated would have limited information on the importance of sports and thus a lower motivation to be active in sports. Contrarily, higher educated hold cultural dispositions that highlight the importance of a healthy mind and body. Consequently, the lower educated feel less urged to participate in sports, while the higher educated consider sports to be more relevant for a healthy and long lasting life.

Second, educational level is associated with the type of work an individual performs. People with a lower educational level often work in more physically demanding jobs (De Breij et al., 2019). Intense physical labour generally is found to be negatively related to sport participation (Mutz et al., 2020). Therefore, the overrepresentation of lower educated people in physically demanding jobs may be a reason for the relatively low sports participation among lower educated people.

Third, people’s social networks may play a role in the extent to which people are stimulated to participate in sports. Based on the homogeneity principle, people with a lower educational level are assumed to have more people with a lower educational level in their core social network (Lin, 2000). Given lower sports participation among lower educated, it is assumed that lower educated are less likely to be stimulated by social network members to participate in sports (Mötteli & Dohle, 2020). Consequently, we expect the lower educated to be at higher risk of not participating in sports compared to higher educated counterparts who are motivated more by their social network to participate in sports.

Finally, the direct living environment of people is associated with education. Education is an important dividing line by which neighbourhoods are clustered. Generally, higher educated live together in residential neighbourhoods with favourable living conditions. In more deprived areas, where lower educated are overrepresented, there is a less activity-friendly environment, and also, the variety of sports facilities is usually more limited (Hoekman et al., 2016). This may thus hinder lower educated to practice sports in the area they live in.

To understand why higher and lower educated have reacted differently to COVID and the restrictions affecting sports participation it seems important to relate this to the above-mentioned factors. Although the importance of sport participation is emphasised by governments during the COVID-pandemic, lower-educated individuals generally are expected to have less knowledge about COVID (Gerosa et al., 2021; Gomes da Silva et al., 2021) and the importance of participating in sports (André et al., 2018; Rademakers, 2014). Therefore, lower educated are expected to feel less urged to find alternative ways to participate in sports during the COVID-pandemic.

Due to the restrictions, working from home became more common during the COVID-pandemic. Higher-educated people are more likely to have a job that can be done from home (Deng et al., 2020). Homework offers additional opportunities to combine it with relaxing sports activity. For instance, it is easier for people working from home, mostly higher educated, to participate in sports at home during their breaks and after working hours.

Another factor relates to people’s social networks. In general, it is important that people are stimulated to become or be active in sports, especially during COVID-times. Mainly, hearing from others about opportunities or alternatives to participate in sports seems an important stimulus (Mötteli & Dohle, 2020). So, shared network experiences with practising sports during the COVID-pandemic may be especially encouraging. Given that the lower educated have a less informed and active network for them, it is less likely to be informed on alternatives and facilities.

Finally, despite COVID-restrictions in the Netherlands, people were allowed to practice outdoor sports individually. In deprived neighbourhoods, with an overrepresentation of lower educated, the environment generally is less physical-activity-friendly (Hoekman et al., 2016). There is relatively limited access to public sports opportunities (e.g. parks, playgrounds, walking and cycling routes), and the perceived safety of the neighbourhood hinders people to participate in sports in the neighbourhood (Van Lenthe et al., 2005).

Summarising, we expect that: *Educational inequality in the level of weekly sport participation increased during the COVID-pandemic.*

### 2.2. Financial deprivation and sport participation

Next to educational factors, it is generally believed that economic deprivation limits the opportunities and chances for people to participate in sports. Financial deprivation may relate to several underlying factors that complicate sport participation. In Bourdieusian terms, financially deprived people are observed as having less economic capital (Bourdieu, 1978). Having limited financial opportunities mostly relates to factors in the domain of working; having no paid labour, flexible labour or a low paying job seriously limits people’s opportunities. Lack of abundant financial resources may be a barrier to participate in sports, since sport participation requires, for example, the purchase of sports equipment, clothing, or a membership fee.

Additionally, participation in sports also entails costs for social commitments. Such social obligations arise from social pressures caused by norms and values within sports. For instance, having a drink after training or taking part in team activities every now and then is seen as part of the deal. Prior research shows that especially financial expenses associated with social obligations are a barrier for people with financial problems to participate in sports (Steenhuis et al., 2009).

Finally, financial deprivation often comes with high levels of stress and worries, and this may be a barrier to participate in sports (Brondolo et al., 2017). Indeed, previous research established that stress is related to reduced physical activity (Stults-Kolehmainen & Sinha, 2014), and we assume this holds true for sport participation as well.

Again, to understand how financial deprivation and sport participation are related to COVID, it is important to account for the above-mentioned factors. In general, we expect that the sport participation of people with financial problems is affected more by the COVID-pandemic. COVID for many people increased uncertainty in the work domain because of (the risks of) becoming unemployed or for flex workers being without clients or orders (Smits et al., 2021). For many people thus financial stress and worries during the COVID-pandemic may contribute to less motivation to practice sports. In general, we therefore assume that increased financial uncertainty in COVID-times results in the avoidance of non-essential financial expenditures, like those on sports. Especially, looking for alternative sports opportunities may come with additional costs that for financially deprived people are less likely to bearable.

Lastly, financial deprivation often comes together with smaller housing, limited outdoor space (e.g. gardens), and less facilities in the neighbourhood. This makes it more difficult to participate in sports at home, which was one of the most popular alternative ways to participate in sports during the COVID-pandemic (Ammar et al., 2020). We thus expect that: *Financial inequality in level of weekly sport participation increased during the COVID-pandemic.*

## 3. Data and methods

To answer our questions, we employed the LISS panel (Scherpenzeel, 2009; see also www.lissdata.nl). This online panel started in 2007 based on a probability sample of households drawn from the Dutch population registers and consisted of approximately 7000 individuals living in 4500 households. Households were provided with a computer and internet access if they did not possess them, and members received financial compensation for participation. Panel attrition was higher among younger and lower educated individuals (Lugtig, 2014), but the LISS panel remained largely representative To answer our questions, we employed the LISS panel (Scherpenzeel, 2009; see also www.lissdata.nl). This online panel started in 2007 based on a probability sample of households drawn from the Dutch population registers and consisted of approximately 7000 individuals living in 4500 households. Households were provided with a computer and internet access if they did not possess them, and members received financial compensation for participation. Panel attrition was higher among younger and lower-educated individuals (Lugtig, 2014), but the LISS panel remained largely representative.

### 3.1. Data source

To test our hypotheses, we use information from the LISS panel executed between 7 June and 27 July 2021. For the purpose of this study, we were able to add questions to the core modules of LISS relating to sport participation before and during the COVID-pandemic. The LISS panel consists of about 5000 households randomly drawn from the Dutch population registers. These households comprised approximately 7500 individuals. For our study, we randomly selected one individual per household. All panel members are aged 16 years or older and have proficiency in Dutch. Low-educated young men are somewhat underrepresented in LISS. Therefore, we have weighted our data for educational level, age, and gender to safeguard representativity. More information about the LISS panel is found at www.lissdata.nl.

Our sample includes respondents with both a valid score on sport participation before the COVID-pandemic, and during COVID-times (*N* = 2.825). We also excluded respondents with missing information on their educational level, financial situation and relevant controls (6.1% missing). Our final sample thus consists of 2.652 respondents.

### 3.2. Measurements

Our survey contains retrospective questions on sport participation in 2019, and in March, April and May 2021. *Level of weekly sport participation* is measured with two questions. First, respondents were asked whether they participated in sports in the last three months (March, April and May 2021), and subsequently whether they participated in sports before COVID (referring to the year 2019). This questioning of sport participation is subjective as respondents were free to interpret sport participation as they understood it. Second, for those who indicated to take part in sports, the number of hours per week (on average) was questioned. We consider measuring weekly sport participation a valid way of indicating an individual’s sport participation, compared to measuring daily, monthly or yearly sport participation. The organisation of people’s sport participation generally follows a week structure, with events or competitions during the weekends and trainings during the weeks. In addition, weekly sport participation is one of the key indicators for sport policy in the Netherlands. For our study, we employed an ordinal measure of hours per week active in sports. People who indicated not to participate in sports score of 0. We acknowledge that the measurement of hours per week may be less precise than minutes per week. Within sport participation research, however, the focus is usually on the frequency of participation, with generally the emphasis on whether or not an individual participates in sports weekly. In our case, the additional information on hours per week is an advancement in most sport participation studies.

*Educational level* is measured by a respondent’s highest level of education with a diploma. Respondents with intermediate secondary education or lower were coded into lower educated (0); with higher secondary education and intermediate vocational education into middle educated (1); and those who completed higher vocational education or university into higher educated (2).

*Financial deprivation* is measured with six items referring to a respondent’s financial situation. Questions referred to having trouble making ends meet, being unable to quickly replace things that break, having lend money for necessary expenditures, running behind in paying rent/mortgage or general utilities, having a debt collector/bailiff at the door, and receiving financial support from family or friends. Answers refer to whether they faced such financial problems in March, April or May 2021. A scale is calculated by a simple count. Since this scale proved very skewed (>85% = 0) we dichotomised it so it refers to having financial problems (at least one of the items answered with ‘yes’) (1) or not (all items answered with ‘no’) (0).

We control for *gender* (0 = female, 1 = male), *age* and *ethnic background* **(**Dutch, non-Western and Western) in our analyses. To ensure that changes in inequality are not related to individual experiences with COVID, we control for having had *infection with COVID.* Respondents who indicate they are/were infected with COVID score 1. We also took into account whether people suffer from *long term health problems or a handicap* (1 = yes, 0 = no). Finally, we have controlled for whether there are *children living at home* (1 = yes, 0 = no) Table 1.

**Table 1.** Descriptives.

	Minimum	Maximum	Mean	Std. dev
Dependent variable				
Sports participation 2021 (hours per week)	0	50	2.135	3.398
Sports participation 2019 (hours per week)	0	60	2.786	3.738
Independent variables				
Educational level				
Lower educated	0	1	0.240	0.427
Middle educated	0	1	0.378	0.485
Higher educated	0	1	0.383	0.486
Financial problems during COVID-pandemic (ref.=no)	0	1	0.126	0.332
Gender (ref.=male)	0	1	0.501	0.500
Age (mean = 0)	−26.9	27.1	0.000	15.466
Ethnic background				
Dutch	0	1	0.778	0.416
Non-Western	0	1	0.121	0.326
Western	0	1	0.101	0.302
Infected with COVID (ref.=no)	0	1	0.140	0.346
Having long-term health problems or handicap (ref.=no)	0	1	0.255	0.436
Having kids living at home (ref.=no)	0	1	0.413	0.492

Source: LISS, 2021, *N = 2.652.*

### 3.3. Analytical strategy

First of all, we present simple descriptive results on experienced changes in the level of sport participation during the COVID-pandemic. To do so, we compare the average level of weekly sport participation in hours per week from 2019 and Spring 2021 separately for educational groups and those who are financially deprived and those who are not.

Next, we perform multivariate OLS regression analyses to test whether the descriptive results about any educational and financial inequality in sport participation hold after controlling for relevant variables. Subsequently, a cross-lagged regression analysis is conducted to further test our hypotheses. An individual’s level of sport participation may change in two directions; a person may sport for less hours per week or completely quit participating, or a person may participate in sports for more hours per week or start participating. To account for these variations we include both participants and non-participants in sports before the COVID-pandemic. As a robustness check, we performed the analyses also with only sports participants included. This effort lead to similar outcomes.

Finally, we have conducted a multinomial logistic regression analysis to test whether there are differences between lower- and higher-educated people and people with and without financial problems in the likelihood to completely quitting participating in sports or participating sports less hours per week during COVID. Having an equal or higher level of weekly sport participation during the COVID-pandemic is the reference category.

## 4. Results

### 4.1. Descriptive results

**Figure 2.**
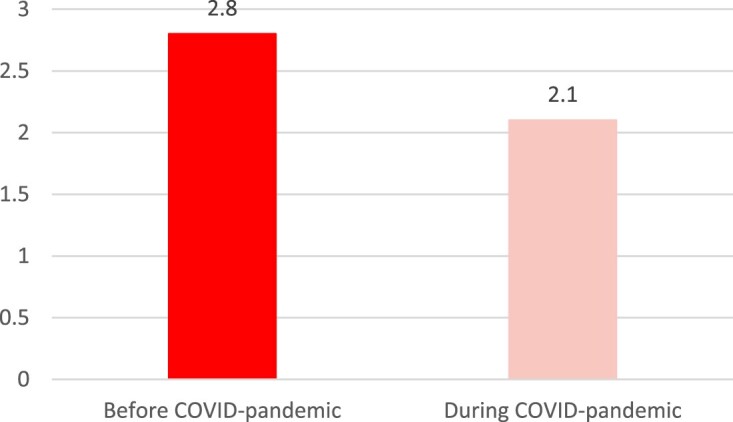
Change in the level of weekly sport participation during the COVID-pandemic.

First, we display whether the average level of weekly sport participation changed during the COVID-pandemic. Figure 2 shows that this indeed is the case. The average number of hours active in sports declined from 2.8 to 2.1 which proved to be significant.

**Figure 3.**
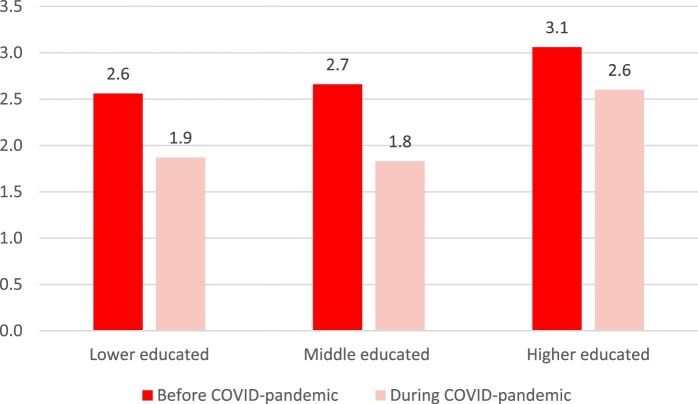
Average level of weekly sport participation by educational level.

**Figure 4.**
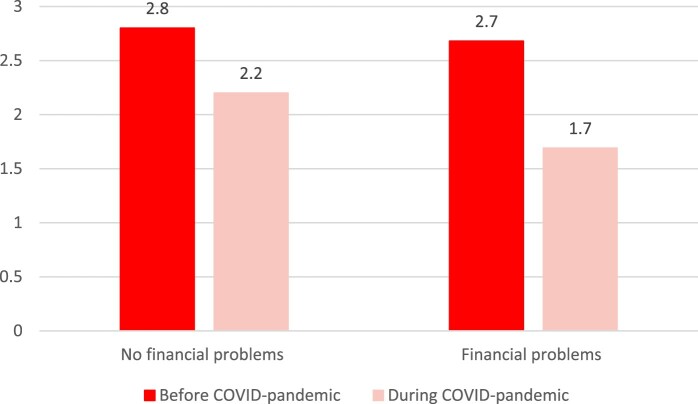
Average level of weekly sport participation by financial situation.

To determine whether there is educational and/or financial inequality in weekly sport participation before and during the COVID-pandemic, descriptive results are presented in Figures 3 and 4. As shown in Figure 3, higher-educated people have a higher level of weekly sport participation (3.1 h before COVID and 2.6 h during COVID) compared to lower- and middle-educated individuals, both before and during the COVID-pandemic. Figure 4 illustrates that the level of weekly sport participation is lower for people with financial problems (2.7 h before COVID and 1.7 h during COVID) compared to people without financial problems, particularly during the COVID-pandemic.

### 4.2. Multivariate results

Table 2 reports the results of our multivariate OLS regression analyses and cross-lagged regression analyses. In model 1, a respondent’s educational level and financial situation are included. It shows that higher educated have a significantly higher level of weekly sport participation during the COVID-pandemic compared to lower-educated people (B = 0.709). No significant difference in the level of weekly sport participation is observed between lower-educated and middle-educated people. Model 1 also shows that financially deprived people report a significantly lower level of weekly sport participation during the COVID-pandemic in comparison to people without financial problems (B = −0.411). Model 2 underscores that educational and financial inequality in weekly sport participation during the COVID-pandemic also exists after controlling for various relevant variables.

**Table 2.** OLS regression analysis predicting sport participation during the COVID-pandemic.

	Model 1		Model 2		Model 3	S.e.
	Estimate	S.e.	Estimate	S.e.	Estimate	
Sports participation 2019 (hours per week)					0.618***	0.013
Educational level (ref.=lower educated)						
Middle educated	−0.044	0.171	−0.065	0.171	−0.110	0.125
Higher educated	0.709***	0.171	0.708***	0.172	0.414***	0.126
Financial problems during COVID-pandemic (ref.=no)	−0.411*	0.199	−0.354*	0.203	−0.277*	0.149
Gender (ref.=male)			0.524***	0.131	0.055	0.097
Age (mean = 0)			−0.015***	0.005	0.002	0.003
Ethnic background (ref.=Dutch)						
Non-Western			−0.651**	0.206	−0.634***	0.151
Western			0.075	0.218	−0.043	0.160
Infected with COVID (ref.=no)			0.149	0.189	−0.028	0.139
Having long-term health problems or handicap (ref.=no)			−0.064	0.157	0.100	0.115
Having kids living at home (ref.=no)			−0.017	0.140	0.141	0.103
Intercept	1.932***	0.138	1.743***	0.180	0.303*	0.136

*p < 0.050, **p < 0.010, ***p < 0.001: two-tailed test.

Source: LISS, 2021, *N* = 2.652.

To test our expectations on possible changes in educational and financial inequality in sport participation during the COVID-pandemic, in model 3, the results of a cross-lagged regression analysis are reported. In this model, to explain the current level of sports participation (during COVID-pandemic), we include a respondent’s level of weekly sport participation before the COVID-pandemic as a predictor, which allows us to observe whether educational and financial inequalities in sport participation have changed. A positive effect in such a cross-lagged model may be interpreted as a smaller decline in the level of weekly sport participation during the COVID-pandemic, whereas a negative effect is interpreted as a larger decline (compared to the general average as referred to in the intercept).

Model 3 indeed displays that the level of weekly sport participation of higher educated people declined less strongly during the COVID-pandemic (B = 0.414) compared to lower educated people. Consequently, the educational gap in the level of weekly sport participation in the Netherlands increased during the COVID-pandemic. These findings are in line with hypothesis 1.

Regarding differences between people with and without financial problems, model 3 shows a similar pattern: the significant negative effect of having financial problems (B = −0.277) indicates that the decline in the level of weekly sport participation during the COVID-pandemic is stronger for financially deprived people compared to people without financial problems. Subsequently, the financial inequality gap in sport participation enlarged during the COVID-pandemic. This is in accordance with hypothesis 2. As a robustness check, we conducted similar analyses including only respondents who already were active in sports before the COVID-pandemic (in 2019) and found similar results with increasing gaps between higher and lower educated and between those financially deprived and not.

One of the control variables also showed significant estimates of respondent’s change in weekly sport participation during the COVID-pandemic as reported in model 3. Inequality in sport participation became larger regarding migration background; the decline seems strongest among non-Western migrants (B = −0.634) compared to natives.

It is important to note that people who decreased their sport participation refer to both people who declined sport participation to less hours, and people who quit participating in sports during the COVID-pandemic at all. Likely, completely quitting sport is considered even more detrimental for both the individual and society than participating in sports for less hours. To investigate whether there are differences in the likelihood of completely quitting and declining sport participation, we conducted a multinomial logistic regression analysis. Table 3 shows a comparison of people that declined and quitted with sports during COVID, with an equal or higher level of weekly sport participation as a reference category. In these analyses logically only people who participated in sports in 2019 have been included. The models in Table 3 clearly indicate that higher educated are less likely to completely quit participating in sports (Exp(B) = 0.546). Also, people with financial problems are more likely to completely quit sporting than people without financial problems (Exp(B) = 1.819). Regarding the likelihood to participate in sports less hours per week during COVID-pandemic, these multinominal logistic regression analyses showed no significant differences between these groups. Linking this back to the beforementioned increase of educational and financial inequality in sports during the COVID-pandemic, it seems that this increased inequality is mainly the result of the fact that lower educated and people with financial problems hold a higher risk of completely quitting participating in sports, as opposed to solely reducing the hours of sport.

**Table 3.** Multinomial logistic regression analysis predicting likelihood to participate in sport less hours per week and likelihood to completely quit participating in a sport during the COVID-pandemic.

	Model 1	S.e.	Exp(B)	Model 2	S.e.	Exp(B)
	Estimate			Estimate		
Participating in sport less hours per week						
Educational level (ref.=lower educated)						
Middle educated	−0.006	0.185	0.994	0.044	0.190	1.045
Higher educated	−0.219	0.177	0.803	−0.140	0.185	0.869
Financial problems during COVID-pandemic (ref.=no)	0.100	0.224	1.105	−0.068	0.231	0.934
Gender (ref.=male)				0.045	0.129	1.046
Age (mean = 0)				−0.014**	0.005	0.986
Ethnic background (ref.=Dutch)						
Non-Western				0.615**	0.215	1.850
Western				0.032	0.217	1.033
Infected with COVID (ref.=no)				0.366*	0.174	1.442
Having long-term health problems or handicap (ref.=no)				−0.199	0.170	0.820
Having kids living at home (ref.=no)				−0.003	0.136	0.997
Intercept	−0.606***	0.152		−0.789***	0.200	
Completely quit participating in sport						
Educational level (ref.=lower educated)						
Middle educated	0.048	0.174	1.050	0.025	0.175	1.025
Higher educated	−0.540**	0.172	0.583	−0.606***	0.176	0.546
Financial problems during COVID-pandemic (ref.=no)	0.687***	0.191	1.988	0.598**	0.199	1.819
Gender (ref.=male)				−0.165	0.126	0.848
Age (mean = 0)				0.003	0.004	0.876
Ethnic background (ref.=Dutch)						
Non-Western				0.743***	0.208	2.102
Western				−0.132	0.219	0.876
Infected with COVID (ref.=no)				−0.175	0.192	0.839
Having long-term health problems or handicap (ref.=no)				−0.124	0.156	0.883
Having kids living at home (ref.=no)				−0.136	0.136	0.873
Intercept	−0.480***	0.144		−0.310*	0.181	
Equal or higher level of weekly sport participation during the COVID-pandemic is reference category						

*p < 0.050, **p < 0.010, ***p < 0.001: two-tailed test.

Source: LISS, 2021, *N* = 1.531.

## 5. Discussion and conclusions

In this contribution, we addressed the development of educational and financial inequality in sport participation in the Netherlands during the COVID-pandemic. We consider this to be an important topic providing information on increased inequalities under COVID. In addition, we build on prior elaborations of scholars on the possible impact of the COVID-pandemic on social inequality in sport participation (Evans et al., 2020). Theoretically, we focus on the unequal distribution of cultural and economic capital and their impact on sport participation patterns, herewith adding to the body of knowledge on stratification in sport participation, particularly in times of COVID.

In this article, we tested the hypotheses that educational and financial inequality in the level of weekly sport participation increased during the COVID-pandemic. In line with prior studies on the impact of global crises on sports (Collins & Haudenhuyse, 2015; Roberts, 2013), our results confirm this idea. In particular, lower educated and people with financial problems seem to be at higher risk of completely quitting their sport participation during the COVID-pandemic. Based on our theoretical assumptions, our results imply that lower educated and those who are financially deprived have less resources to uphold weekly sport participation. Given the importance of practising sport and the beforementioned obstacles in upholding this participation, it seems important that these obstacles are mitigated or removed wherever possible. Our study reaffirms the importance of cultural and economic capital in studying sport participation (Demarest et al., 2014; European Commission, 2017; Hoekman et al., 2017). Our main conclusion drawn from the results is that educational and financial inequality in the level of weekly sport participation increased during the COVID-pandemic. We consider this to be an important result, amongst others because sports participation is associated with a lower risk of severe COVID-outcomes when infected (Halabchi et al., 2021).

Despite the current attention to sports to achieve a society in which people are more resilient to health problems, the average level of sport participation during COVID-times decreased in the Netherlands, as well as in other EU member states (Council of Europe, 2020; Mutz & Gerke, 2021; NOC*NSF, 2020; Tison et al., 2020). Generally, this decrease is attributed to COVID-measures and related restrictions. Although COVID-policy measures apply to everyone, our findings indicate that some groups are affected more than others. Contradicting findings to this were particularly found in studies at the beginning of the lockdowns, with more severe restrictions for sports participation (Bu et al., 2021; Mutz & Gerke, 2021). In the spring of 2021 in the Netherlands, only some small restrictions regarding sports were still in play, with additional possibilities to re-start sports activities or to find alternative ways to practice sports. Consequently, our study highlights that lower educated and people with financial problems are less likely to bounce back to their original sport participation after the strict lockdown measures and face more problems to pick sport participation up again after the peak of the COVID-pandemic. This confirms our theoretical assumptions that these groups likely have relatively few resources (e.g. social contacts, knowledge and money) to adapt to the COVID-measures and find alternatives (to continue) to participate in sports.

A clear limitation of our study is that it focuses on a period that still held some COVID-restrictions. Consequently, a follow-up study is necessary for 2022, when COVID-measures no longer restrict possibilities to practice sports. Furthermore, we questioned people on whether they practiced sports. It is however possible that respondents were still a member of a sports club, but were, due to COVID restrictions, not actively practising this sport at the period of questioning. Third, we are aware that self-reported data may suffer from social desirability and random error because of memory bias. Since we ask questions about (sport) behaviour and not about opinions and attitudes, we however believe that our self-reported data on sport participation are less susceptible to social desirability. A final drawback of our research is that lower-educated youngsters are somewhat underrepresented in our data. We have tried to deal with this issue by weighing for age and educational level. Nevertheless, it should be noted that in the LISS panel, similar to other web panels and sport participation surveys specifically (Bethlehem, 2010; Haudenhuyse, 2018), vulnerable groups are underrepresented (Knoef & Vos, 2008). This could imply that our results of increased inequality only show the top of the iceberg, as the more vulnerable groups, who might be impacted the most, are less sufficiently represented in the panel data.

A few implications of our study may be noted. First, our findings may inform policymakers to critically assess their sport promotion policies. Our results evidently underscore that the current policy attention is not sufficient in achieving an inclusive sports sector (Ministerie van VWS, 2018). Consequently, there is a need to intensify policy attention to address the observed growing inequality due to the COVID pandemic. By doing so it is important to not solely focus on sports-promoting policies when aiming to stimulate people to participate in sports. It is crucial to address underlying issues related to the basic needs of individuals. To illustrate, people who cannot make ends meet and have no food on the table likely will not practice sports even when stimulated to. Before being successful in stimulating these people to participate in sports, financial uncertainty needs to be combatted. Available funds in the Netherlands that cover the costs of sport participation for those in poverty are helpful in this regard. Together with these funds, the Neighbourhood Sport Coaches (NSC), as front-line professionals (Hoogendam, 2021), can play an important role. NSCs are the core of sport promotion policies in the Netherlands with a strong focus on more vulnerable groups. During the COVID-pandemic, however, half of the NSC report to be less active in organising activities to stimulate sport participation among these groups due to other tasks related to the COVID-measures (Ooms & Van Stam, 2021). It could be that this contributed in some way to the increased inequality that we found in our study. This brings us to our second implication. We were able to distillate the inequality within the Netherlands, but not the processes behind the increased inequality. The limited activities of NSC could be one reason, but other factors could be at play as well. To better address inequality, insight is needed into the motives and barriers of people from vulnerable groups, the role of sport in their daily lives, and how COVID impacted this. While our study helps to identify ‘who’ lags behind in sport participation, qualitative research may help to identify more specifically ‘why’ lower educated and people with financial problems lag behind compared to higher educated and those with no financial problems and ‘how’ these groups can be activated. Third, we anticipate that the findings of increased inequality are also applicable to other countries. Considering the low wage differences and social inequality in the Netherlands, compared to international standards, and the national and local policy discourse on the value and importance of sport (Hoekman et al., 2022; Hoekman & Breedveld, 2013), it is likely that the impact of COVID on sport participation might be larger in other European countries. Therefore, more specific country studies on the effects of the COVID-pandemic on sport participation are recommended. This could in addition to more general studies and overviews of sport participation in Europe (European Commission, 2017; Scheerder et al., 2011) add to the understanding of social stratification in sport participation in Europe. This is particularly relevant in the current context of the Sustainable Development Goals, with an emphasis on equality and access to sports (Council of Europe, 2021). Finally, it is important to note that among sport policymakers, there seems to be a ‘pervasive and nearly unshakable belief in the inherent purity and goodness of sport’ (Coakley, 2015, p. 403), referred to as the ‘Great Sport Myth’. Several sports sociologists have disputed this approach of sport as a panacea for all societal problems (e.g. Coalter, 1998, 2007; Elling, 2017; Haudenhuyse, 2018). We acknowledge that sport is not the solution to all negative consequences of the COVID-pandemic on various aspects of life (e.g. work and financial position).

To conclude, our study enhances the understanding of inequalities in the level of weekly sport participation in times of the COVID-pandemic and may inform policymakers to intensify sport promotion policies. Ongoing attention to specific target groups is required to reduce inequality in sport participation. The current COVID-pandemic illustrates clearly how vulnerable sport participation is of lower educated and people with financial problems.

## Note

1 Weekly sport participation is deemed to be an adequate measure for regular sport participation and is a key indicator of sport policy in the Netherlands (see www.sportenbewegenincijfers.nl/kernindicatoren).
